# Nitric oxide regulates phagocytosis through S-nitrosylation of Rab5

**DOI:** 10.1016/j.jbc.2025.110696

**Published:** 2025-09-08

**Authors:** Makoto Hagiwara, Hiroyuki Tada, Kenji Matsushita

**Affiliations:** 1 Department of Health and Nutrition, Faculty of Human Life Studies, University of Niigata Prefecture, Niigata-city, Niigata, Japan; 2 Division of Oral Immunology, Tohoku University Graduate School of Dentistry, Sendai, Miyagi, Japan; 3 Department of Dental Hygiene, Ogaki Women’s College, Ogaki-city, Gifu, Japan

**Keywords:** Rab5, nitric oxide, phagocytosis, S-nitrosylation, small GTPase, redox reaction, membrane trafficking, infection, lipopolysaccharide, immunity, phagocytic cells, iNOS, cysteine residues, GEF-like action, prenylation-independent

## Abstract

Phagocytosis is mediated mainly by immune cells, such as macrophages, monocytes, and neutrophils, which clear large pathogens including bacteria. The small GTP-binding protein Rab5 is crucial for both clathrin-dependent endocytosis and phagocytosis, but the role and mechanism of Rab5 activation during phagocytosis are poorly understood. Here, we report that nitric oxide (NO), a novel regulator of Rab5, regulates phagocytosis through S-nitrosylation of Rab5. NO can promote phagocytosis by activating Rab5 in cultured cells, and it potently S-nitrosylates active Rab5 compared to inactive Rab5. Moreover, we demonstrate that two cysteine residues in the C terminus of Rab5 are S-nitrosylated and are important for phagocytosis. Experiments involving mice showed that NO activates Rab5, increases levels of S-nitrosylated Rab5 and is involved in phagocytic bacterial clearance mediated by peritoneal macrophages. Together these data suggest that NO promotes S-nitrosylation of Rab5 to act as a novel Rab5 activator and a key regulator of phagocytosis.

Phagocytosis in mammals is carried out mainly by immune cells such as macrophages, monocytes and neutrophils, which function to clear large pathogens including bacteria, or large debris such as the remnants of dead cells or arterial fat deposits (1, 2). Phagocytosis is defined as the cellular engulfment of particles ≥0.5 μm in diameter and is a highly regulated process that involves cell surface receptors (including cell adhesion proteins), intracellular signal transduction, actin remodeling, and membrane trafficking (1, 2). For instance, binding of cellular receptors to bacteria triggers intracellular signaling, resulting in remodeling of the actin cytoskeleton and engulfment of bacteria by cell membranes that form the phagocytic cup. Subsequently, the leading edge of the growing cup closes, and a membrane vesicle (phagosome) develops (1, 2). Phagosomes containing bacteria undergo acidification and mature from Rab5-positive early to Rab7-positive late stages (2, 3, 4, 5). Last, the phagosomes fuse with lysosomes to form phagolysosomes that degrade bacteria (2, 6, 7). However, many aspects of the mechanisms by which Rab5 regulates phagocytosis remain unclear.

Rab proteins are small GTPases that regulate vesicular transport in endocytosis and exocytosis (8, 9, 10). To date, over 60 distinct Rab proteins have been identified, and each protein is specifically associated with a particular organelle or pathway (8, 9, 10). Rab5, a member of the Rab family of GTPases, localizes to the plasma membrane and to endosomes (10, 11, 12, 13, 14, 15, 16, 17, 18, 19). Several processes (*e.g.*, budding, trafficking, tethering, and fusion of early endosomes) in eukaryotic cells require Rab5 for the coordination of endocytosis including phagocytosis that occurs *via* specific interactions involving over 30 effector proteins (2, 8, 9, 10, 20, 21). For example, Rabaptin-5 (22, 23), EEA1 (24), APPL1 (25, 26, 27), Rabankyrin-5 (28), and caveolin-1 (29, 30, 31) are known effectors of Rab5, and each protein is involved in regulation of endocytosis through interactions with Rab5. Cycling of Rab5 between an active (GTP-bound) and an inactive (GDP-bound) conformation is regulated by guanine nucleotide exchange factors (GEFs), GTPase-activating proteins (GAPs), and Rab GDP dissociation inhibitor (8, 9, 10). Rab5 replaces GDP with GTP through interactions with various GEF proteins such as Rabex-5 (23), Rin1 (32), Rin2 (33), Rin3 (34) and Alsin (35, 36) that recognize specific residues in the switch regions of Rab5 to facilitate GDP release. Conversion from the Rab5 GTP-bound form to the Rab5 GDP-bound form arises through GTP hydrolysis, which is controlled by the inherent GTPase activity of Rab5 but also by GAPs such as RabGAP-5 (37), RN-tre (38), Armus (TBC-2) (39, 40), TBC1D17 (41), and TBC1D18 (42). Inactivated Rab5 separates from early endosome membranes (and also from the early phagosome membrane) and is retained in the cytosol by Rab GDP dissociation inhibitor until the next round of the GTPase cycle begins (8, 9, 10). Posttranslational modifications of Rab5 also affect its activity. Rab5 has long been known to undergo C-terminal geranylgeranylation that is thought to promote Rab5 membrane binding (9, 10, 43, 44, 45, 46). Rab5 phosphorylation mediated by PKCε has been shown to be involved in regulating cell migration (47, 48). Mono-ubiquitination of Rab5 inhibits its effector binding and guanine nucleotide conversion (49). In this manner, posttranslational modifications cause dynamic changes in Rab5 function.

Nitric oxide (NO) plays significant roles in various physiological processes including the regulation of immune responses (50), signal transduction (51, 52, 53), and blood pressure regulation (54). NO is produced from L-arginine by the NO synthase (NOS) isoforms neuronal NOS (nNOS, or NOS1), inducible NOS (iNOS, or NOS2), and endothelial NOS (eNOS or NOS3) in cells (52, 55). Recent studies reported that addition of a NO moiety (NO•) to cysteines, a process called S-nitrosylation, represents a key signaling pathway that regulates protein functions (56, 57, 58). Protein S-nitrosylation is related to eNOS, nNOS, and iNOS expression levels in several tissues and is restricted to regions of the cell where NOS localizes (59, 60, 61). In addition, protein S-nitrosylation can be analyzed experimentally using NO donors such as S-nitrosoglutathione (GSNO), an agent that generates NO (62, 63). Many proteins—including caspase-3 (64), MyD88 (65), beta-actin (66), caveolin-1 (67), and PTEN (68, 69)—are reported to be S-nitrosylated. We previously reported that S-nitrosylation of N-ethylmaleimide-sensitive factor, an ATPase that is essential for activating membrane fusion machinery, regulates exocytosis of Weibel–Palade bodies and platelet granule (70, 71). However, the impact of NO on regulatory factors that control endocytosis and phagocytosis remains unclear. In this study, we describe a novel molecular mechanism by which NO regulates phagocytosis through S-nitrosylation of Rab5.

## Results

### NO regulates phagocytosis

**Figure 1:**
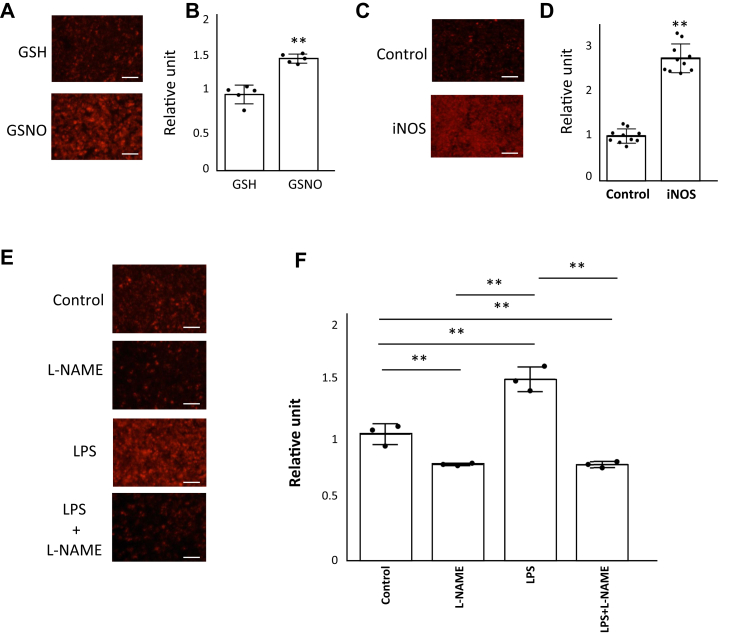
**NO regulates phagocytosis.***A* and *B*, RAW264 cells were incubated with 100 μM GSNO, a NO donor, or GSH for 1 h. The cells were then incubated with pHrodo Red *S. aureus* BioParticle conjugates for phagocytosis for 1 h. *A*, images were taken with a fluorescence microscope. The scale bar represents 200 μm. *B*, values measured with a fluorescent microplate reader. Each value in the graph is the mean ± SD of five independent experiments. ∗∗*p* < 0.01. *C and D*, RAW264 cells transfected with an iNOS vector or a control vector were incubated for 1 h 37 °C with pHrodo Red *S. aureus* BioParticle conjugates for phagocytosis. *C*, images taken with a fluorescence microscope. The scale bar represents 200 μm. *D*, values measured with a fluorescent microplate reader. Each value in the graph is the mean ± SD of 10 independent experiments. ∗∗*p* < 0.01. *E* and *F*, RAW264 cells were incubated with 1 mM L-NAME, 100 ng/ml LPS, 100 ng/ml + L-NAME, or dimethyl sulfoxide (DMSO) alone (control) for 24 h. The cells were then incubated with pHrodo Red *S. aureus* BioParticle conjugates for phagocytosis. *E*, images taken with a fluorescence microscope. The scale bar represents 200 μm. *F*, values measured with a fluorescent microplate reader. Each value in the graph is the mean ± SD of three independent experiments. ∗∗*p* < 0.01. GSNO, S-nitrosoglutathione; iNOS, inducible nitric oxide synthase; L-NAME, NG-Nitro-L-arginine methyl ester; LPS, lipopolysaccharide; NO, nitric oxide.

We first examined the effect of NO on phagocytosis in macrophage-like RAW264 cells. GSNO is a source of NO (NO donor) and an important mediator of NO signaling. GSNO can generate NO through processes such as heat, photodecomposition, metal ions, reducing agents, and other mechanisms. Here, we used GSNO to investigate the effect of NO on phagocytosis. RAW264 cells were preincubated with GSNO or GSH (control) for 1 h and the uptake of pHrodo Red *Staphylococcus aureus* BioParticles, a marker of phagocytosis, was measured. RAW264 cells incubated with GSNO had increased phagocytic uptake compared to those incubated with GSH (Fig. 1, *A* and *B*). Phagocytosis was also upregulated in RAW264 cells with overexpression of iNOS (Fig. 1, *C* and *D*). Stimulation with lipopolysaccharide (LPS) is known to dramatically increase iNOS expression and NO production in cells (72, 73). To clarify whether LPS-stimulated NO production promotes phagocytosis, we conducted experiments using NG-Nitro-L-arginine methyl ester (L-NAME), a nonselective NOS inhibitor. The results showed that stimulation with LPS alone for 24 h promoted phagocytosis, whereas phagocytosis was decreased in RAW264 cells treated with L-NAME alone or with both LPS and L-NAME for 24 h (Fig. 1, *E* and *F*). Taken together, these results suggest that NO upregulates phagocytosis in RAW264 cells.

### iNOS interacts with Rab5

**Figure 2:**
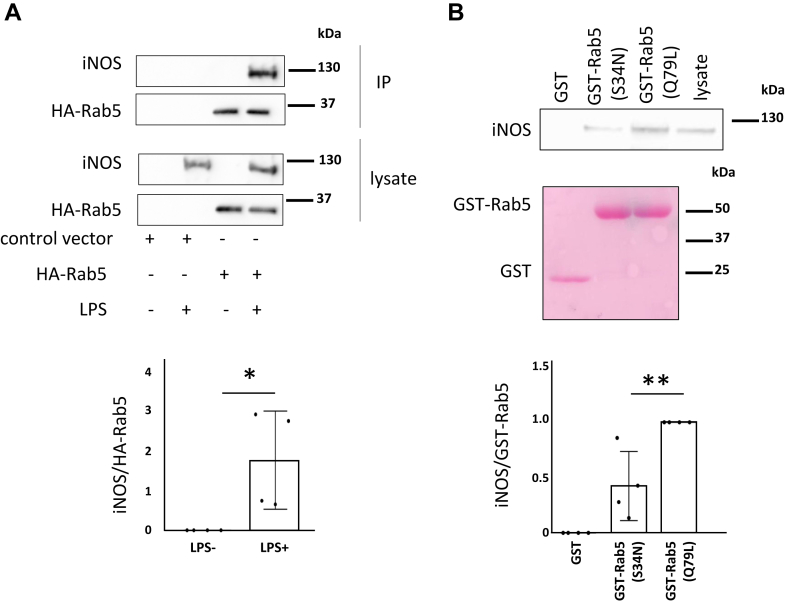
**iNOS interacts with Rab5.***A*, RAW264 cells transfected with HA-Rab5 or control vector were incubated with 100 ng/ml LPS for 16 h. HA-Rab5 in lysates from LPS-stimulated RAW264 cell lysates was then immunoprecipitated and iNOS binding to Rab5 was assessed with Western blotting (*top panel*). Quantification of iNOS/HA-Rab5 (*lower panel*). Each value in the graph is the mean ± SD of four independent experiments. ∗*p* < 0.05. *B*, A pull-down assay with LPS-stimulated RAW264 cell lysates was performed using GST, GST-Rab5(S34N), and GST-Rab5(Q79L). Binding of iNOS to the beads was assayed with Western blotting (*top panel*). GST, GST-Rab5(S34N), and GST-Rab5(Q79L) on the poly vinylidene difluoride membrane were stained with Ponceau S (*middle panel*). Quantification of iNOS/GST-Rab5 (*lower panel*). Each value in the graph is the mean ± SD of four independent experiments. ∗*p* < 0.01. GST, glutathione-*S*-transferase; iNOS, inducible nitric oxide synthase; L-NAME, NG-Nitro-L-arginine methyl ester; LPS, lipopolysaccharide.

Next, we examined whether iNOS interacts with Rab5. RAW264 cells transfected with HA-Rab5 or control vector were incubated with or without 100 ng/ml LPS for 16 h. iNOS was coimmunoprecipitated with HA-Rab5 in lysates from LPS-stimulated RAW264 cells (Fig. 2*A*). To investigate whether iNOS interacts with inactive and/or active Rab5, we performed a glutathione-*S*-transferase (GST) pull-down assay using lysates from LPS-stimulated RAW264 cells. The results revealed that iNOS binds more strongly to active Rab5 mutant (Q79L) than to inactive Rab5 mutant (S34N) (Fig. 2*B*). These data suggest that iNOS interacts with Rab5.

### NO directly increases Rab5 activity

**Figure 3:**
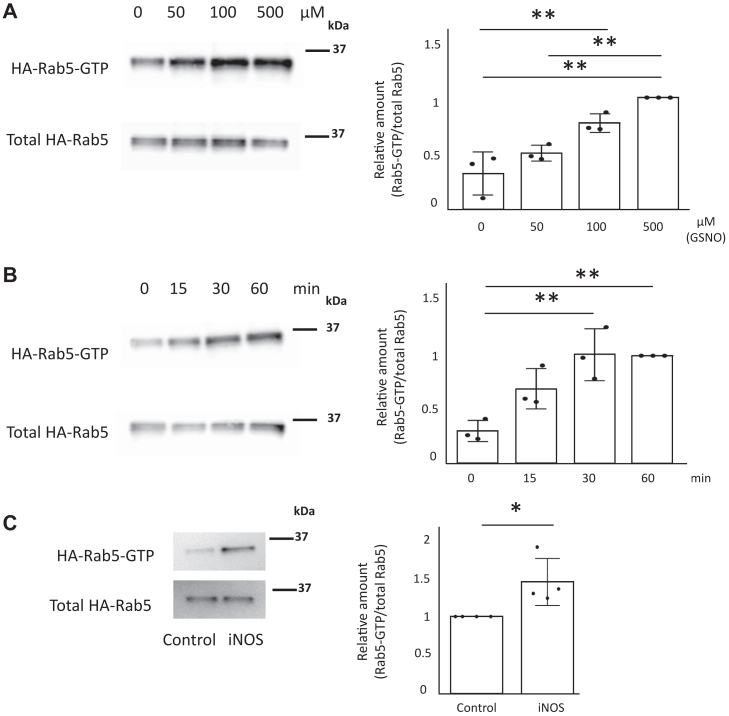
**NO increases Rab5 activity.***A*, HEK293 cells transfected with an HA-Rab5 vector were incubated with 0 to 500 μM GSNO for 60 min. Rab5-GTP was assessed with a GST-R5BD pull-down assay. Each value in the graph is the mean ± SD of three independent experiments. ∗∗*p* < 0.01. *B*, HEK293 cells transfected with an HA-Rab5 vector was incubated with 100 μM GSNO for 0 to 60 min Rab5-GTP was assessed with a GST-R5BD pull-down assay. Each value in the graph is the mean ± SD of three independent experiments. ∗∗*p* < 0.01. *C*, HEK293 cells were transfected with iNOS and HA-Rab5 vectors and Rab5-GTP was assessed with a GST-R5BD pull-down assay. Each value in the graph is the mean ± SD of four independent experiments. ∗*p* < 0.05. GSNO, S-nitrosoglutathione; GST, glutathione-S-transferase; iNOS, inducible nitric oxide synthase; L-NAME, NG-Nitro-L-arginine methyl ester; NO, nitric oxide.

**Figure 4:**
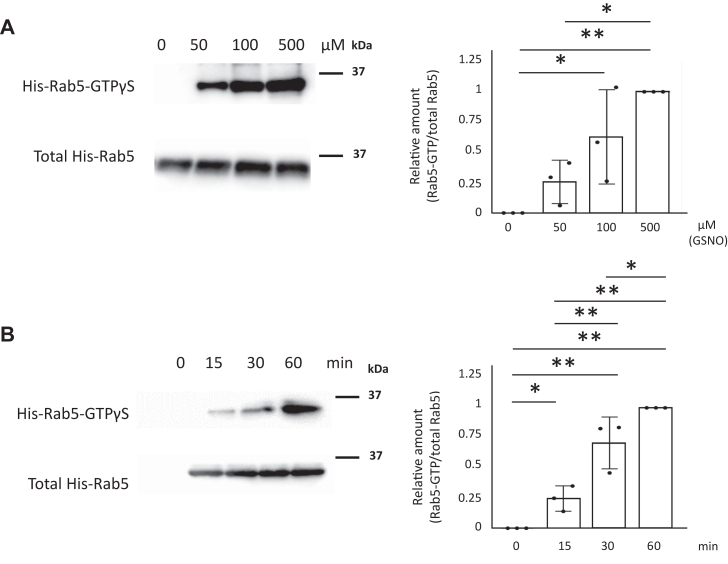
**NO directly increases Rab5 activity.***A*, purified recombinant His-Rab5 (wild) was incubated with 0 to 500 μM GSNO and GTPγS for 30 min at room temperature and Rab5-GTPγS was assessed with a GST-R5BD pull-down assay. Each value in the graph is the mean ± SD of three independent experiments. ∗*p* < 0.05 and ∗∗*p* < 0.01. *B*, purified recombinant His-Rab5 (Wild) was incubated with 100 μM GSNO and GTPγS for 0 to 60 min at room temperature, and Rab5-GTPγS was assessed with a GST-R5BD pull-down assay. Each value in the graph is the mean ± SD of three independent experiments. ∗*p* < 0.05 and ∗∗*p* < 0.01. GSNO, S-nitrosoglutathione; NO, nitric oxide.

Since NO activated phagocytosis (Fig. 1, *A*–*F*), we hypothesized that NO would also promote Rab5 activity. We used a Rabaptin-5 Rab5-binding domain–based GST pull-down assay (GST-R5BD pull-down assay) to evaluate Rab5 activation (74). We found that Rab5 activity in HEK293 cells dose and time dependently increased in response to GSNO (Fig. 3, *A* and *B*). Moreover, HEK293 cells expressing iNOS and HA-Rab5 exhibited increased Rab5 activity (Fig. 3*C*). To further examine whether NO directly enhanced Rab5 activity, we used purified recombinant His-Rab5. Again we found that GSNO dose and time dependently increased activity of purified Rab5 (Fig. 4, *A* and *B*). These data suggested that NO indeed regulates Rab5 activity.

**Figure 5:**
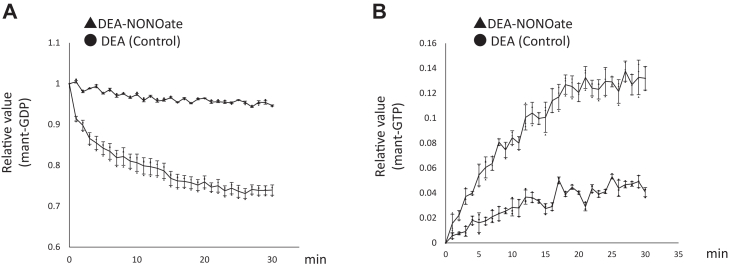
**NO is a guanine nucleotide exchange factor for Rab5.***A*, mant-GDP-loaded recombinant His-Rab5 was incubated with DEA-NONOate. The fluorescence emission was monitored for 30 min. Each value in the graph is the mean ± SD of three independent experiments. *B*, recombinant His-Rab5 was incubated with DEA-NONOate and mant-GTP. The fluorescence emission was monitored for 30 min. Each value in the graph is the mean ± SD of three independent experiments. DEA-NONOate, diethylamine NONOate; NO, nitric oxid.

We next analyzed the Rab5-mediated GDP/GTP nucleotide exchange reaction in response to NO. Aqueous solutions of GSNO have a pink color, which interferes with fluorescence measurements taken with a microplate reader. Therefore, we also used diethylamine NONOate (DEA-NONOate), an NO donor that is colorless in solution and has a short half-life during which NO is rapidly released. Mant-GDP (fluorescent labeled GDP analog)-loaded recombinant His-Rab5 was incubated with DEA-NONOate. DEA-NONOate treatment promoted GDP dissociation from Rab5 (Fig. 5*A*). Meanwhile, incubation of Rab5 with mant-GTP, a fluorescent GTP nucleotide analog, and DEA-NONOate resulted in binding of GTP to Rab5 (Fig. 5*B*). These results indicate that NO is directly involved in the GDP/GTP exchange reaction of Rab5.

### NO S-nitrosylates Rab5

**Figure 6:**
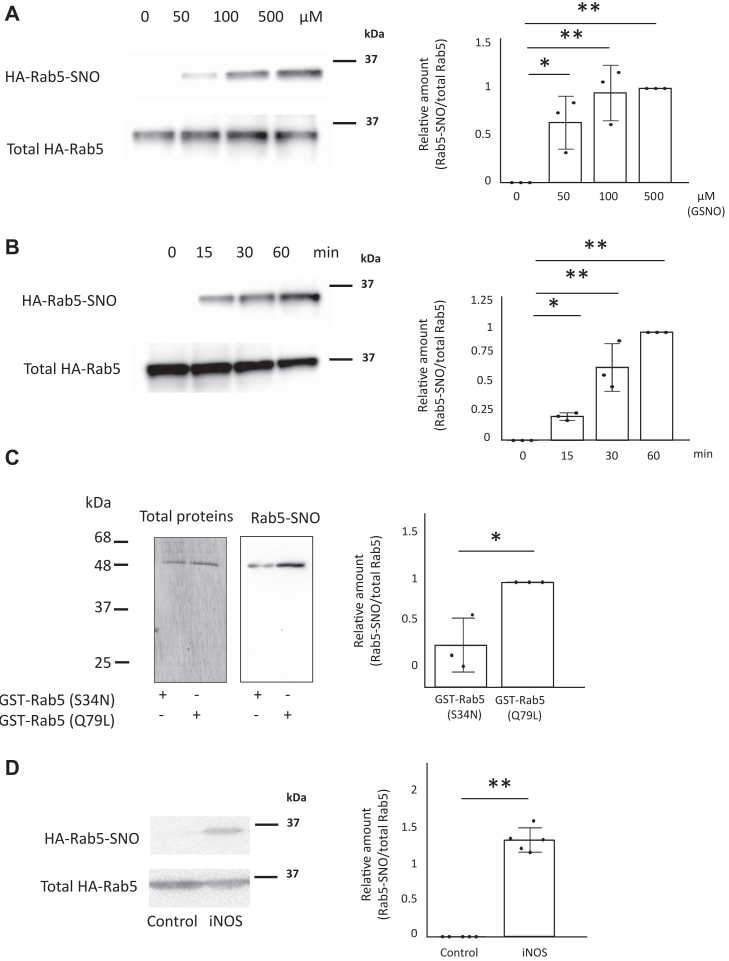
**Rab5 is nitrosylated.***A*, HEK293 cells transfected with an HA-Rab5 vector were incubated with 0 to 500 μM GSNO for 60 min. S-nitrosylation of Rab5 was assessed using a biotin-switch method. Each value in the graph is the mean ± SD of three independent experiments. ∗*p* < 0.05 and ∗∗*p* < 0.01. *B*, HEK293 cells transfected with an HA-Rab5 vector were incubated with 100 μM GSNO for 0 to 60 min. S-Nitrosylation of Rab5 was assessed using a biotin-switch method. Each value in the graph is the mean ± SD of three independent experiments. ∗*p* < 0.05 and ∗∗*p* < 0.01. *C*, purified recombinant GST-Rab5(Q79L) or GST-Rab5(S34N) were incubated with 500 μM GSNO at room temperature for 60 min, and S-nitrosylation was assessed with a biotin-switch method. Each value in the graph is the mean ± SD of three independent experiments. ∗*p* < 0.05. *D*, HEK293 cells were transfected with iNOS and HA-Rab5 vectors and the S-nitrosylation of Rab5 was assessed using a biotin-switch method. Each value in the graph is the mean ± SD of five independent experiments. ∗∗*p* < 0.01. GSNO, S-nitrosoglutathione; GST, glutathione-*S*-transferase; iNOS, inducible nitric oxide synthase.

The biotin-switch assay is a powerful tool for detecting S-nitrosylated proteins (75). We next measured S-nitrosylation of Rab5. HEK293 cells transfected with an HA-Rab5 vector were incubated with GSNO and Rab5 S-nitrosylation was assessed using a biotin-switch assay. Increased S-nitrosylation of Rab5 expressed in HEK293 cells occurred dose and time dependently following GSNO treatment (Fig. 6, *A* and *B*). To investigate whether S-nitrosylation is related to the activity status of Rab5, we detected S-nitrosylation of active Rab5 and inactive Rab5 mutants. Purified GST-Rab5 (Q79L) and GST-Rab5 (S34N) were incubated with GSNO and S-nitrosylation of Rab5 was assessed using a biotin-switch assay. Both active Rab5 (Q79L) and inactive Rab5 (S34N) were S-nitrosylated, but the levels of S-nitrosylation were higher for active Rab5 (Q79L) than for inactive Rab5 (S34N) (Fig. 6*C*). In addition, HA-Rab5 showed increased S-nitrosylation in iNOS- and HA-Rab5–expressing HEK293 cells (Fig. 6*D*). These data suggested that Rab5 is S-nitrosylated by NO.

### S-nitrosylation of Rab5 is reversible

**Figure 7:**
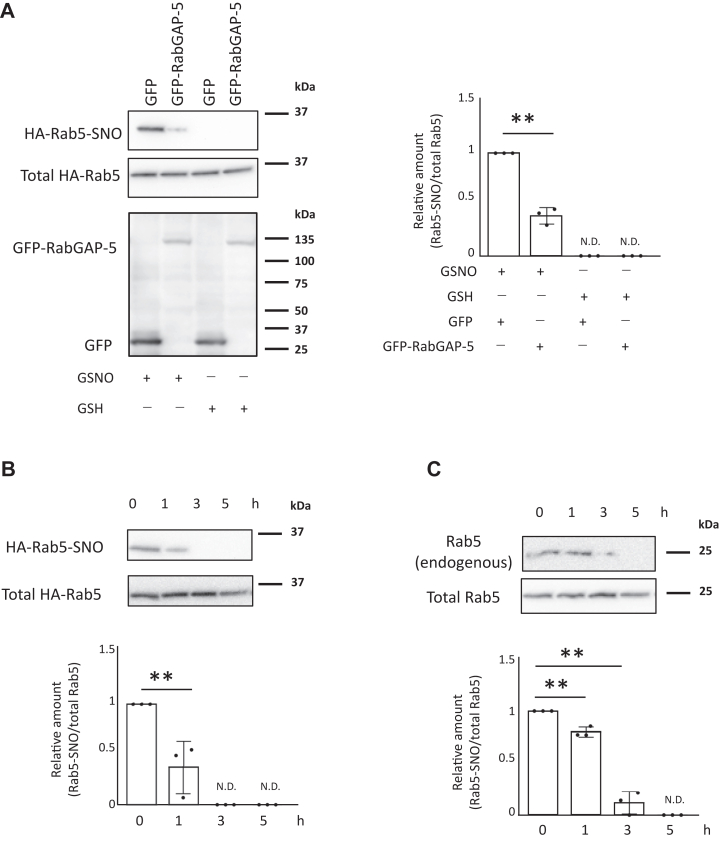
**Rab5 S-nitrosylation is reversible.***A*, HEK293 cells transfected with HA-Rab5 and GFP-RabGAP-5 vectors were incubated with 100 μM GSNO for 60 min. S-Nitrosylation of Rab5 was assessed using the biotin-switch method. Each value in the graph is the mean ± SD of three independent experiments. N.D., not detected. ∗∗*p* < 0.01. *B*, S-Nitrosylation of Rab5 decreases over time in HEK293 cells. HEK293 cells transfected with an HA-Rab5 vector were incubated with 100 μM GSNO for 60 min and then washed. After 0 to 5 h of recovery, cell lysates were harvested and levels of HA-Rab5-SNO were measured with a biotin-switch assay. After exposure to an NO donor, the amount of S-nitrosylated Rab5 decreases over time. Each value in the graph is the mean ± SD of three independent experiments, N.D., not detected. ∗∗*p* < 0.01. *C*, endogenous S-nitrosylation of Rab5 decreases over time. RAW264 cells were incubated with 100 ng/ml LPS for 24 h. After incubation with LPS, RAW264 cells were incubated with 1 mM L-NAME for 0 to 5 h, and then levels of S-nitrosylation of Rab5 were measured with the biotin-switch assay. After NO synthase is inhibited by L-NAME, the amount of endogenous S-nitrosylated Rab5 decreases over time. Each value in the graph is the mean ± SD of three independent experiments, N.D., not detected. ∗∗*p* < 0.01. GSNO, S-nitrosoglutathione; L-NAME, NG-Nitro-L-arginine methyl ester; LPS, lipopolysaccharide; NO, nitric oxide.

To clarify how the Rab5 inactivation factor GAP affects Rab5 S-nitrosylation, we analyzed HEK293 cells expressing GFP-RabGAP-5 (a GAP for Rab5) and HA-Rab5 in a biotin-switch assay. Cells expressing GFP-RabGAP-5 had decreased Rab5 S-nitrosylation relative to control cells expressing GFP only, suggesting that S-nitrosylation of Rab5 is negatively regulated by its GAP (Fig. 7*A*). Next, we examined whether Rab5 S-nitrosylation is reversible. HEK293 cells expressing HA-Rab5 were incubated with GSNO for 60 min, after which the medium was replaced with GSNO-free medium, and denitrosylation was analyzed using a biotin-switch assay. S-nitrosylation of Rab5 decreased over time in these HEK293 cells (Fig. 7*B*). We also used a biotin-switch assay to analyze S-nitrosylation and denitrosylation of endogenous Rab5 in LPS-stimulated RAW264 cells. Endogenous Rab5 was S-nitrosylated 24 h after LPS stimulation. When the medium was replaced 24 h after LPS stimulation with medium lacking LPS and containing 1 mM L-NAME, an NOS inhibitor, the level of S-nitrosylated endogenous Rab5 in RAW264 cells gradually decreased over time. These results indicate that Rab5 S-nitrosylation is reversible.

### S-nitrosylation of Rab5 cysteine residues

**Figure 8:**
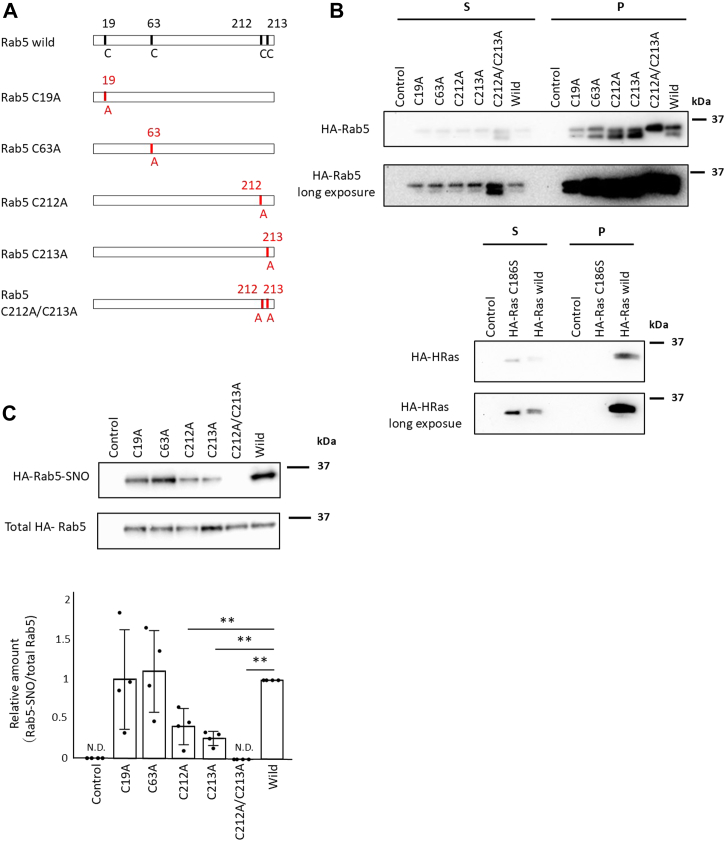

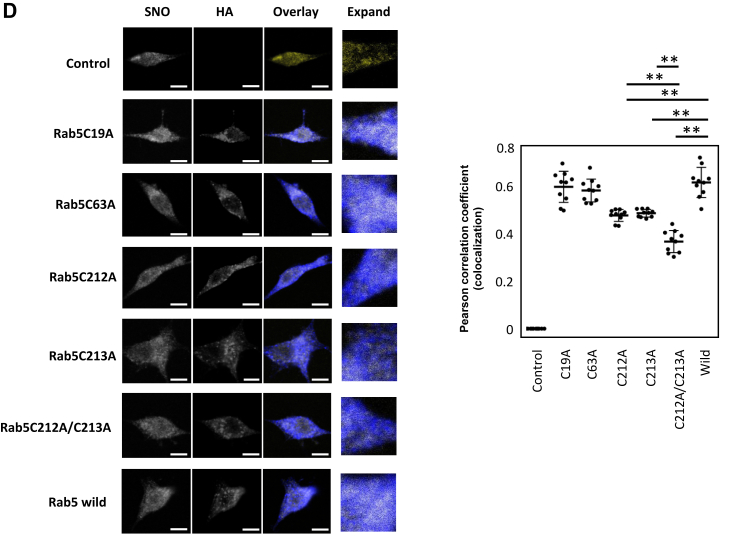
**Rab5 cysteine residues C212 and C213 are S-nitrosylated.***A*, schematic of Rab5 mutants. *B*, HEK293 cells transfected with the indicated HA-Rab5 mutant vector, HA-Rab5 WT vector, or control vector were homogenized and the cell lysates were ultracentrifuged. The resulting supernatant (S) and pellet (P) fractions were analyzed by Western blotting (*top panel*). HEK293 cells transfected with the indicated HA-Ras mutant vector, HA-Ras WT vector or control vector were homogenized and the cell lysates were ultracentrifuged. The resulting supernatant (S) and pellet (P) fractions were analyzed by Western blotting (*lower panel*). *C*, HEK293 cells transfected with the indicated HA-Rab5 mutant vector, HA-Rab5 WT vector, or control vector were incubated with 100 μM GSNO for 1 h. S-Nitrosylation of HA-Rab5 was detected by a biotin-switch assay. Rab5-SNO levels were normalized to total HA-Rab5 levels and quantified using ImageJ. The graph shows the mean ± SE of four independent experiments, ∗∗*p* < 0.01 *versus* HA-Rab5 wild-expressing cells; N.D., not detected. *D*, RAW264 cells transfected with the indicated HA-Rab5 mutant vector, HA-Rab5 WT vector, or control vector. The cells were incubated with 100 ng/ml LPS for 18 h and then fixed with paraformaldehyde. S-Nitrosylated proteins were visualized with a biotin-switch assay using fluorescein-conjugated avidin, and HA-Rab5 was visualized by immunostaining using an anti-HA antibody (primary antibody) and a cy3-conjugated antibody (secondary). The scale bar represents 5 μm. Colocalization of the indicated proteins was quantified with the Pearson correlation coefficient method of pixel intensity correlation measurements using ImageJ Fiji software (*right panel*). Each value in the graph is the mean ± SD of 10 cells. ∗∗*p* < 0.01. GSNO, S-nitrosoglutathione; NO, nitric oxide; LPS, lipopolysaccharide.

After demonstrating that Rab5 is S-nitrosylated (Figs. 6 and 7), we next identified the S-nitrosylation sites. S-nitrosylation has consistently been reported to occur at cysteine residues (56, 76, 77). The Rab5 amino acid sequence in mice has four cysteine residues: C19, C63, C212, and C213. We mutated these cysteine residues to alanine, which does not undergo S-nitrosylation, and also created mutants in which the two cysteine residues on the C-terminal side were mutated to alanine. (Fig. 8*A*). To analyze the subcellular distribution of each HA-Rab5 mutant, the cytosolic (S) and membrane-containing particulate (P) fractions were prepared by ultracentrifugation of cell lysates. All HA-Rab5 variants, including HA-Rab5C212A/C213233wA (double mutation of Rab5 with two C-terminal cysteines mutated to alanine), were detected in both the S and P fractions (Fig. 8*B*). HA-HRasC1866S was not detected in the P fraction upon fractionation as confirmed using a HRas C-terminal cysteine mutant that is not detected in the P fraction as a positive control (Fig. 8*B*). This result was unexpected given that previous reports showed that Rab5 binds membranes *via* lipid modifications at the cysteines in the C-terminal of Rab5 (see Discussion).

### Cysteine residues 212 and 213 in Rab5 are important for Rab5 activity and phagocytic activity

We used HA-Rab5 mutants in a biotin-switch assay to identify the S-nitrosylation site of Rab5 mediated by NO. HEK293 cells transfected with the indicated HA-Rab5 mutant vectors, HA-Rab5 WT vector or a control vector were incubated with 100 μM GSNO for 1 h. For HA-Rab5C212A and HA-Rab5C213A mutant proteins, low levels of Rab5 S-nitrosylation were detectable by Western blotting of lysates from GSNO-treated cells, but S-nitrosylation was completely absent in cells expressing the HA-Rab5C212A/C213A double mutant (Fig. 8*C*). S-nitrosylated proteins can also be detected by confocal fluorescence microscopy using the biotin-switch assay with fluorescently labeled avidin (60, 78, 79). Here, RAW264 cells transfected with the indicated HA-Rab5 mutant vectors, HA-Rab5 WT vector or control vector were incubated with LPS for 18 h, and then the subcellular localization of S-nitrosylated proteins and HA-Rab5 was assessed using a biotin-switch assay and immunostaining. Confocal fluorescence microscopy revealed that the single mutants HA-Rab5C212A and HA-Rab5C213A, as well as the double mutant HA-Rab5C212A/C213A, had reduced colocalization with S-nitrosylated protein signals compared to HA-Rab5 WT (Fig. 8*D*). Notably, the colocalization of the HA-Rab5 C212A/C213A double mutant with S-nitrosylated protein signals was reduced compared to the colocalization seen for either the HA-Rab5C212A and HA-Rab5C213A single mutants (Fig. 8*D*). Determination of a Pearson’s correlation coefficient between S-nitrosylated proteins and Rab5 mutants in the confocal fluorescence microscopy analyses demonstrated a trend that was consistent with the Western blot analysis of S-nitrosylated Rab5. Taken together, these results indicate that the two C-terminal cysteine residues, Cys 212 and Cys213, of Rab5 are crucial for S-nitrosylation.

**Figure 9:**
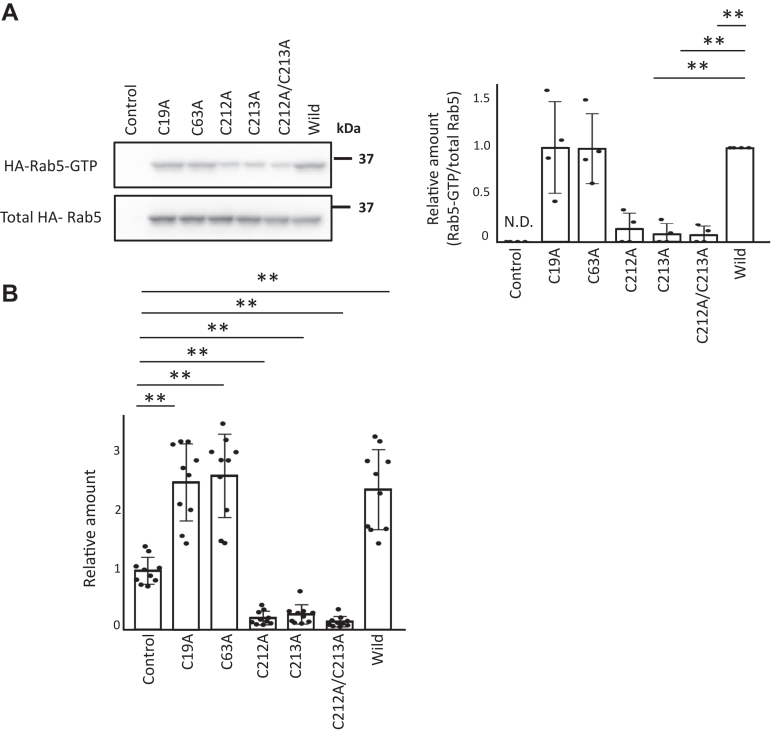
**Rab5 cysteine residues C212 and C213 are important for activity and phagocytic activity.***A*, HEK293 cells transfected with the indicated HA-Rab5 mutant vector, HA-Rab5 WT vector, or control vector were incubated with GSNO for 1 h. Rab5 activity was then assessed with a GST-R5BD pull-down assay. Each value in the graph is the mean ± SD of four independent experiments. ∗∗*p* < 0.01 *versus* Rab5 WT, N.D., not detected. *B*, RAW264 cells transfected with the indicated HA-Rab5 mutant vector, HA-Rab5 WT vector, or control vector were incubated with pHrodo *Red S. aureus* BioParticle conjugates for phagocytosis for 1 h. Phagocytosis of *S. aureus* was measured using a fluorescence microplate reader. Each value in the graph is the mean ± SD of 10 independent experiments. ∗∗*p* < 0.01 *versus* vector (mock)-transfected cells. GSNO, S-nitrosoglutathione; GST, glutathione-*S*-transferase.

To clarify whether activation of Rab5 by NO involves S-nitrosylation of Rab5, HEK293 cells transfected with the HA-Rab5 C212A and C213A single mutant vectors, HA-Rab5C212A/C213 vector, HA-Rab5 WT vector, or control vector were incubated with GSNO for 1 h and Rab5 activity was measured in a GST-R5BD pull-down assay. Compared to WT HA-Rab5, HA-Rab5C212A, HA-Rab5C213A, and HA-Rab5C212A/C213A mutants, all had lower Rab5 activity (Fig. 9*A*). Next, we examined the effects of each HA-Rab5 mutant on phagocytosis to establish a relationship between Rab5 S-nitrosylation and phagocytosis. Overexpression of HA-Rab5C212A, HA-Rab5C213A, or HA-Rab5C212A/C213A in RAW264 cells significantly reduced phagocytic activity compared to that seen for HA-Rab5 WT (Fig. 9*B*). These results again indicate that the C-terminal cysteine residues C212 and C213 in Rab5 are important for Rab5 activity and phagocytosis.

### Two C-terminal cysteines of Rab5 induce formation of phagosomes

**Figure 10:**
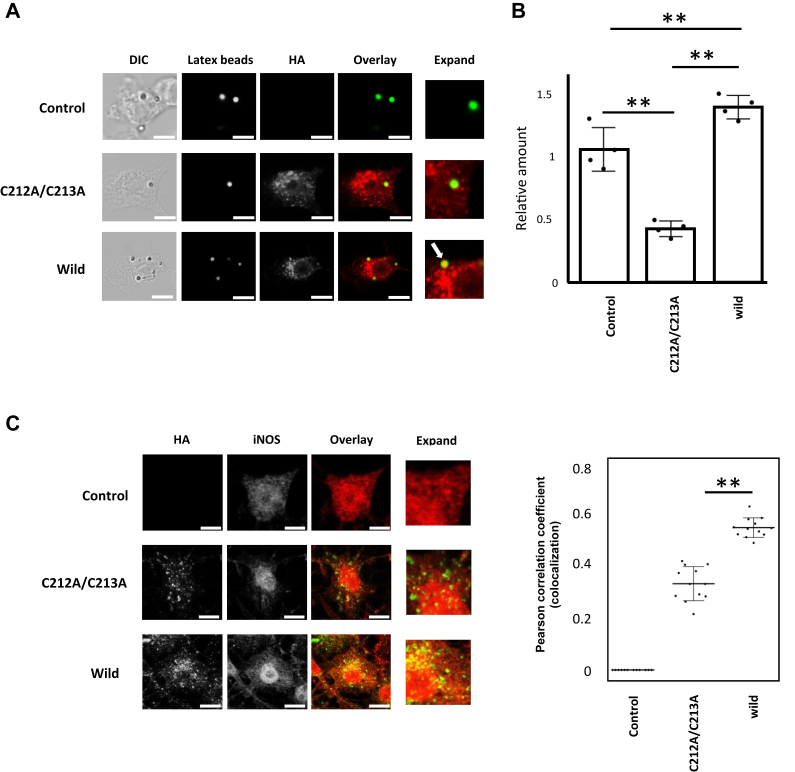
**Two C-terminal cysteines of Rab5 induce formation of phagosomes.***A*, RAW264 cells expressing either HA-Rab5 WT or HA-Rab5 C212A/C213A were incubated with 100 ng/ml LPS for 24 h, followed by the addition of fluorescent latex beads to observe phagosome formation. The scale bar represents 5 μm. *B*, RAW264 cells expressing either HA-Rab5 WT or HA-Rab5 C212A/C213A were incubated with 100 ng/ml LPS for 24 h before fluorescent latex beads were added. Bead uptake was then quantified. Each value in the graph is the mean ± SD of four independent experiments. ∗∗*p* < 0.01 *C*, RAW264 cells expressing either HA-Rab5 WT or HA-Rab5 C212A/C213A were incubated with 100 ng/ml LPS for 24 h and the subcellular localization of HA-Rab5 or HA-Rab5C212A/C213A and iNOS was then examined (*left panel*). The scale bar represents 5 μm. Colocalization of the indicated proteins was quantified using the Pearson correlation coefficient method of pixel intensity correlation measurements with ImageJ Fiji software (*right panel*). Each value in the graph is the mean ± SD of 12 cells. ∗∗*p* < 0.01. iNOS, inducible nitric oxide synthase; LPS, lipopolysaccharide.

To analyze the ability of S-nitrosylated Rab5 to promote phagosome formation, we used confocal fluorescence microscopy to observe phagocytosis of latex beads added to LPS-stimulated RAW264 cells. HA-Rab5 WT induced formation of phagosomes in LPS-stimulated RAW264 cells, but HA-Rab5C212A/C213 did not (Fig. 10*A*). Furthermore, phagocytosis of latex beads was significantly reduced in LPS-stimulated cells expressing the HA-Rab5C212A/C213A mutant compared to those expressing WT Rab5 (Fig. 10*B*). The HA-Rab5C212A/C213A mutant also had reduced colocalization with iNOS relative to HA-Rab5 WT (Fig. 10*C*). Together, these results suggest that S-nitrosylation of the two C-terminal cysteine residues of Rab5 is important for formation of phagosomes.

### Effect of NO on Rab5 and *S. aureus* clearance *in vivo*

We further analyzed Rab5 activity and Rab5 S-nitrosylation using LPS, a potent inducer of iNOS that results in high levels of NO production *in vivo*. Mice received tail vein injections with 4 μg/g weight LPS or 4 μg/g weight + 0.1 mg/g weight L-NAME. The mice were sacrificed 24 h later and the spleens were collected. Cytoplasmic fractions of the spleen were prepared and analyzed for Rab5 activity by a GST-R5BD pull-down assay and Rab5 S-nitrosylation was assessed with a biotin-switch assay.

**Figure 11:**
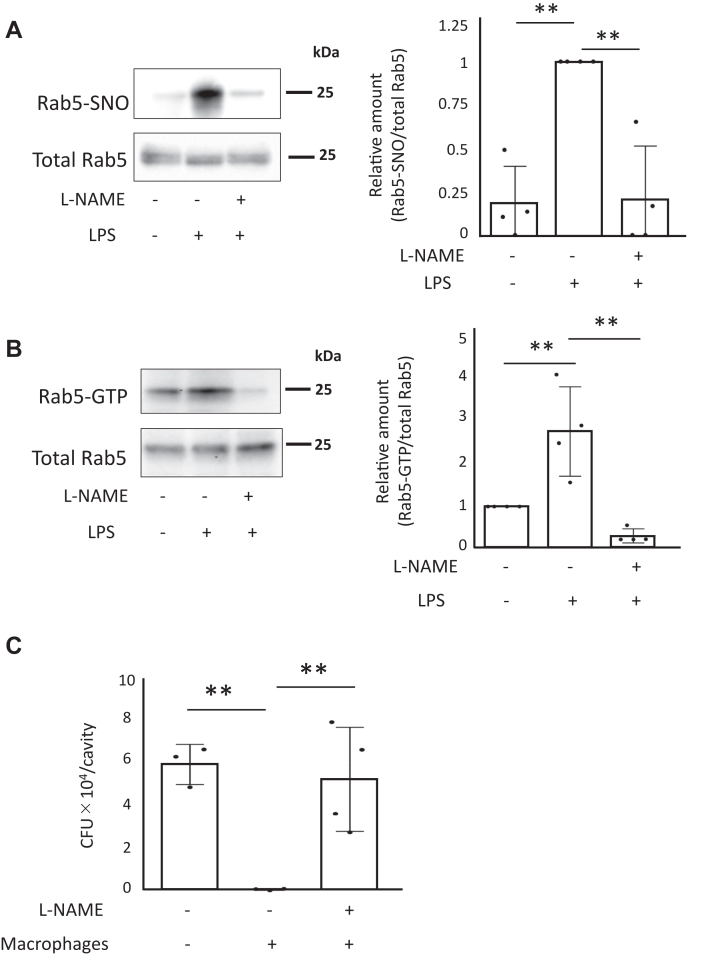
**Effect of NO on Rab5 and phagocytosis *in vivo*.** Mice were injected intravenously with LPS or LPS and L-NAME and sacrificed 24 h later. The spleens were removed and homogenized. The homogenates were analyzed for (*A*) Rab5 S-nitrosylation using a biotin-switch assay and (*B*) Rab5 activity by GST-R5BD pulldown. Values in the graphs are the mean ± SD of four independent experiments. ∗∗*p* < 0.01. *C*, murine peritoneal macrophages were pretreated with 1 mM L-NAME for 24 h. Eight-week-old BALB/c mice were injected intraperitoneally with L-NAME-treated 4 × 10^6^ CFU of murine peritoneal macrophages and 3 × 10^9^ CFU of *S. aureus* ATCC25923. After 6 h, *S. aureus* bacteria were collected by peritoneal lavage and plated on tryptic soy agar. CFU values are the mean ± SD for 3 to 4 mice. ∗∗*p* < 0.01. CFU, colony-forming unit; GSNO, S-nitrosoglutathione; NO, nitric oxide; GST, glutathione-*S*-transferase; L-NAME, NG-Nitro-L-arginine methyl ester; LPS, lipopolysaccharide.

**Figure 12:**
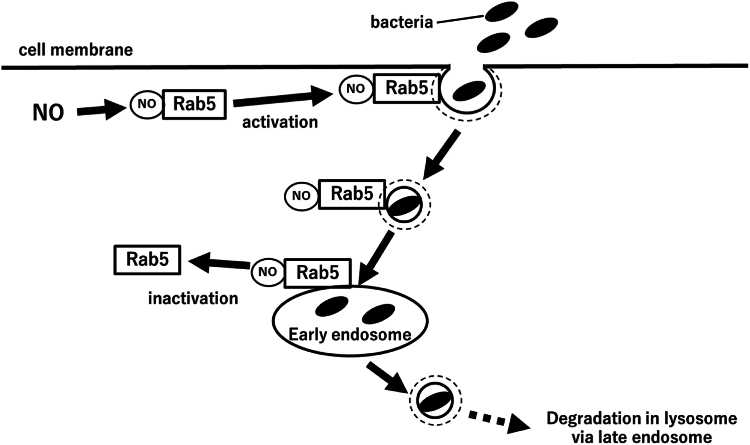
**Model for the role of Rab5 in S-nitrosylation-mediated phagocytosis.** Rab5 is activated by NO-mediated S-nitrosylation and promotes phagocytosis. Internalized materials such as bacteria are transported to early endosomes (early phagosomes) by S-nitrosylated Rab5. The results of this study and previous studies together suggest that bacteria would be transported from early endosomes (early phagosomes) *via* late endosomes to lysosomes by Rab7. The bacteria are then degraded in the lysosomes. NO, nitric oxide.

The GST-R5BD pull-down assay showed increased Rab5 activity in mice treated with LPS alone compared to untreated mice, while Rab5 activity was increased in mice treated with LPS and L-NAME compared to untreated mice (Fig. 11*A*). Biotin-switch assays indicated enhanced Rab5 S-nitrosylation in mice treated with LPS alone compared to untreated mice, and attenuated S-nitrosylation of Rab5 in mice treated with LPS and L-NAME compared to untreated mice (Fig. 11*B*). Mouse peritoneal macrophages were also treated with L-NAME for 24 h and then the treated macrophages (5 × 10^6^) were injected into 8-week-old BALB/c mice together with 3 × 10^9^ colony-forming unit (CFU) of *S. aureus* ATCC25923. After 6 h, *S. aureus* were collected by peritoneal lavage and plated on tryptic soy agar. The colonies were enumerated as CFUs. Determination of CFUs showed that the number of viable bacteria in untreated cells was very low (6.4 × 10 CFUs), whereas the number of viable bacteria in the L-NAME–treated cells was very high (5.4 × 10^4^ CFUs) (Fig. 11*C*). The *in vivo* experimental results, together with the results from cultured cells and *in vitro* experiments, suggest that NO-mediated regulation of Rab5 has a physiologically important role in phagocytosis (Fig. 12).

## Discussion

In the present study, we demonstrate that NO directly activates Rab5 and enhances phagocytosis. Previous reports showed that GEF activates Rab5, but to our knowledge, this is the first study to show that NO activates Rab5 in the absence of traditional GEF. Moreover, our data newly uncover that phagocytosis is activated by NO, indicating that NO may regulate phagocytosis independently of geranylgeranylation by S-nitrosylating Rab5.

Rab5 is reported to play a role both in clathrin-dependent endocytosis and phagocytosis. In the present study, NO activated Rab5 and promoted phagocytosis (Fig. 1, *A*–*F*). Within cells, levels of NO produced by iNOS are known to be markedly elevated during bacterial infection (80). NO is known to have direct bactericidal effects, but at levels that are substantially higher than those present under physiological conditions (50). In particular, NO concentrations decrease with increasing distance from iNOS (50). Instead, phagocytosis, in which bacteria are taken up by endocytosis and degraded, is a more effective means to eliminate bacteria during bacterial infection. Previous reports showed that NO activates phagocytes that in turn promote phagocytosis (81, 82), although the detailed molecular mechanisms of this activation are largely unclear. Our results are consistent with previous reports showing that NO activates phagocytosis in phagocytic cells, and we further demonstrated that NO activates Rab5 in phagocytosis.

Protein S-nitrosylation is related to expression levels of eNOS, nNOS, and iNOS in tissues (83, 84) and is restricted to regions of the cell where NOS is localized (59, 60). The production of NO from arginine mediated by iNOS activates macrophages (81, 82), which are essential for host defenses against many pathogens. During phagocytosis, iNOS can be recruited to phagosomes containing *Escherichia coli* (85), *Listeria monocytogenes* (86), or latex beads in interferon- and LPS-stimulated macrophages (87, 88). Our results show that iNOS expression increases phagocytosis (Fig. 1, *C* and *D*) and that Rab5 interacts with iNOS (Fig. 2, *A* and *B*). Moreover, Rab5 is S-nitrosylated in the presence of iNOS expression (Fig. 6*D*). Based on these observations, Rab5 S-nitrosylation may be enhanced in regions of the cell, where iNOS localizes to promote phagocytosis.

NO was previously shown to directly affect NO-mediated GDP/GTP exchange activity of the small GTP-binding protein Ras (89, 90). Dynamin, the founding member of a family of dynamin-like GTPases, is implicated in membrane fission and remodeling and has a critical role in endocytic membrane fission (91). Like Ras, the activity of dynamin-2 is directly regulated by NO through S-nitrosylation (92, 93, 94). Here, we found that Rab5 is also S-nitrosylated and its GDP/GTP exchange is directly controlled by NO (Figs. 4, *A*, *B* and 5, *A*, *B*). The three-dimensional structures of inactivated (GDP-binding form) and activated small GTP-binding proteins (GTP-binding form) differ (95). Together, these findings suggest that NO may regulate the function of proteins through S-nitrosylation that promotes structural changes that have a direct activating effect on GTP-binding proteins and other G proteins (*e.g.*, dynamin).

The C terminus of many small GTP-binding proteins is prenylated by the attachment of geranylgeranyl or farnesyl groups (95). In Rab5, the C-terminal residues C212 and C213 undergo posttranslational geranylgeranyl modification that is thought to be important for plasma membrane targeting of Rab5 (45, 96). Here, we show that NO activates Rab5 and that the C212 and C213 cysteine residues of Rab5 are S-nitrosylated by NO (Fig. 8, *C* and *D*), suggesting that Rab5 may bind to membranes in a prenylation-independent manner. Rab13, another Rab family member, is reported to undergo geranylgeranylation of a C-terminal cysteine residue (97). However, Ioannou *et al.* reported that Rab13 lacking the C-terminal geranylgeranyl binding site still binds to membranes and proposed a model in which Rab13 binds to membranes *via* protein–protein interactions (98). Meanwhile, the C terminus of Ras undergoes farnesylation that is thought to be important for membrane binding (99, 100). Ras is also reported to be activated and membrane-bound in a prenylation-independent manner (101). Thus, prenylation is important for membrane binding of small GTP-binding proteins, but this membrane binding can also occur in a prenylation-independent manner. Like Rab13, Rab5 with C-terminal S-nitrosylation may also bind to membranes through protein–protein interactions. Alternatively, multiple small GTPases including Rab5 (29, 30, 102), Rab3 (103), Rap1 (102, 104), and Ras (102) are reported to localize and bind to lipid rafts (detergent-resistant membrane fraction) that are enriched in cholesterol and glycophospholipids (105). A large number of proteins have been found in the detergent-insoluble lipid raft fraction and are thought to bind strongly to lipid rafts through protein–lipid interactions (105). The mechanism by which Rab5 binds to membranes in a geranylgeranylation-independent manner is a subject for future study.

In Western blotting, the P fraction of cells expressing the HA-Rab5C212A/C213A mutant showed only one band compared to the two bands detected for Rab5 WT and other mutants (Fig. 8*B*). Previous reports showed that prenylated small GTP-binding proteins migrate more slowly on SDS-PAGE than do unprenylated small GTP-binding proteins (101, 106). As such, for Western blotting of the HA-Rab5C212A/C213A mutant only one band corresponding to the unprenylated protein would be expected, whereas Rab5 WT and the other mutants would be expected to have two bands corresponding to prenylated and nonprenylated protein. On the other hand, in the C fraction, two bands were detected for Rab5C212A/C213A, whereas only one band was detected for Rab5 WT and other mutants (Fig. 8*B*). The reason for this difference is unclear, but there is extensive evidence for band shifts in Western blotting that exist when a protein undergoes certain posttranslational modifications. We speculate that posttranslational modifications of the Rab5C212A/C213A mutant and Rab5 WT or other mutants may be different.

In this study, we demonstrated that cysteine residues at positions 212 and 213 in the C-terminal region of Rab5 undergo S-nitrosylation. However, these two cysteine residues are also known to be sites of geranylgeranylation. Therefore, it is necessary to explain how Rab5 can be S-nitrosylated under these conditions. To date, no protein de-geranylgeranylase has been identified, and thus Rab5 that has undergone geranylgeranylation is generally considered to be irreversibly modified. Taking this into account, we propose that S-nitrosylation occurs on newly synthesized, nongeranylgeranylated Rab5 (see below).

As mentioned above, previous studies reported that prenylated small GTPases migrate more slowly on SDS-PAGE compared to their nonprenylated counterparts (102, 107). In our data as well, two bands corresponding to endogenous Rab5 were detected by Western blotting (Fig. 8*B*). As such, between the two bands shown in Figure 8*B*, the band with the slightly lower molecular weight is predicted to represent nongeranylgeranylated Rab5, indicating the presence of endogenous nonprenylated Rab5. Although geranylgeranylation is considered to be an irreversible modification, the presence of nongeranylgeranylated Rab5 in cells suggests that such Rab5 can undergo S-nitrosylation. For example, under inflammatory or infectious conditions in which macrophages or leukocytes express iNOS and produce large amounts of NO, newly synthesized Rab5 (nongeranylgeranylated) may undergo S-nitrosylation. Moreover, our data demonstrate that endogenous Rab5 undergoes S-nitrosylation both in cultured cells (Fig. 7*C*) and *in vivo* (Fig. 11*A*), indicating that this modification would occur under physiological conditions and is not merely an artifact of overexpression.

We also considered the conditions under which Rab5 is more likely to undergo S-nitrosylation. As described above, the presence of nonprenylated endogenous Rab5 suggests that an increase in total Rab5 expression would also lead to an increase in the amount of nonprenylated Rab5. Therefore, increased Rab5 expression may enhance the susceptibility of nongeranylgeranylated Rab5 to S-nitrosylation. Upregulation of Rab5 expression has been reported in the brains of aged animals and in individuals with Alzheimer's disease (107). In addition, increased Rab5 expression has been observed in J774E cells (mouse macrophages) treated with the proinflammatory cytokine interleukin-6 (108), in the placentas of severe acute respiratory syndrome 2 **(**SARS-CoV-2)–positive patients (109, 110), and in cultured cells exposed to particulate matter 2.5 (PM2.5; particles with an aerodynamic diameter <2.5 μm) in combination with SARS-CoV-2 (111). Aging (112), Alzheimer’s disease (113), inflammation (114), and SARS-CoV-2 infection (115) are all associated with elevated expression of iNOS, which leads to increased (NO production. When the expression of both Rab5 and iNOS is increased, Rab5 may be more susceptible to S-nitrosylation.

In terms of other conditions under which Rab5 may be more susceptible to S-nitrosylation, geranylgeranylation of Rab5 is known to involve Rab escort protein (REP) and Rab geranylgeranyl transferase (RabGGTase) (9). Newly synthesized, non-geranylgeranylated Rab proteins first bind to REP, after which the Rab–REP complex interacts with RabGGTase to catalyze Rab5 geranylgeranylation (9). Therefore, a decrease in REP expression or impairment of its function could influence Rab5 S-nitrosylation. Previous studies reported that REP dysfunction, which is associated with retinal dystrophy, is associated with reduced geranylgeranylation of Rab proteins (116, 117). Although the precise mechanisms are still being investigated, REP deficiency or malfunction is believed to inhibit RabGGTase-dependent Rab geranylgeranylation. Moreover, in inherited retinal degenerative diseases, iNOS expression is elevated and NO production is increased (118). Thus, in inherited retinal degenerative diseases in which REP function is impaired, nongeranylgeranylated Rab5 may accumulate and Rab5 could be more likely to undergo iNOS-mediated S-nitrosylation.

Furthermore, other conditions may also promote S-nitrosylation of Rab5. Geranylgeranyl diphosphate (GGPP), which is essential for geranylgeranyl modification, is an intermediate in the cholesterol biosynthesis pathway. A decrease in GGPP production would be expected to increase levels of nongeranylgeranylated Rab5. For example, in female hypercholesterolemic mice, expression of geranylgeranyl diphosphate synthase is markedly reduced in oocytes, and this reduced expression has been shown to affect the function of Rab proteins (119). Similarly, patients with severe obesity as well as mice that had long-term high-fat diet feeding were reported to have reduced geranylgeranyl diphosphate synthase expression that would likely lead to decreased GGPP synthesis (120). Clinically, statins are widely prescribed for patients with hypercholesterolemia, and experimentally are used as inhibitors of protein geranylgeranylation (121). Treatment with statins increases levels of nongeranylgeranylated Rab5 (121, 122). Statins inhibit hydroxymethylglutaryl-CoA reductase, a key enzyme in the cholesterol biosynthesis pathway, thereby reducing the production of downstream metabolites, including GGPP, which plays a crucial role in protein geranylgeranylation and particularly geranylgeranylation of Rab proteins (123). Thus, a reduction in GGPP levels *via* inhibition of the cholesterol biosynthesis pathway would increase the amount of nongeranylgeranylated Rab5 in turn making Rab5 more susceptible to S-nitrosylation.

In the present study, we found that NO activates phagocytosis, promotes S-nitrosylation of Rab5, and increases Rab5 activity *in vivo* (Fig. 11, *A*–*C*). These *in vivo* results (Fig. 11) together with the findings from *in vitro* studies and cultured cells (Figure 1, Figure 2, Figure 3, Figure 4, Figure 5, Figure 6, Figure 7, Figure 8, Figure 9, Figure 10), suggest that NO plays a role in regulating phagocytosis *via* Rab5, which is part of immune system function in living organisms. Phagocytosis is important for removal of bacteria (traditional phagocytosis) (1, 2) and dead cells from organisms (recently termed efferocytosis) (124, 125). Increased expression of Rab5 in alveolar macrophages was recently reported to enhance efferocytosis, suggesting that Rab5 may be a critical factor in this process (126). Abnormalities in removal of dead cells due to decreased phagocytosis can lead to autoimmune disease and chronic inflammatory disease (124, 127). Phagocytosis is also reported to play a role in eliminating cancer cells and in neurological diseases such as Alzheimer's disease and (128, 129, 130, 131). Thus, S-nitrosylation of Rab5 could be involved in a variety of diseases beyond just infectious diseases.

In conclusion, here, we revealed that NO causes S-nitrosylation of Rab5 and plays a crucial role in the regulation of Rab5 function in phagocytosis. Furthermore, the results suggest that S-nitrosylated Rab5 may regulate phagocytosis in a geranylgeranylation-independent manner. To our knowledge, our study is the first to show that Rab proteins are S-nitrosylated in mammalian cells; it is possible that Rab proteins other than Rab5 are also S-nitrosylated. Further clarification of the relationship between NO and Rab proteins will provide new insight into membrane trafficking and various pathological mechanisms involving these small GTPase proteins. Importantly, this study demonstrates that the C-terminal cysteine residue of Rab5 undergoes a modification distinct from geranylgeranylation, highlighting the possibility that the C-terminal cysteine residues of Rab proteins and other small GTPases may be subject to nonprenyl modifications.

## Experimental procedures

### Cell culture

RAW264 cells (RIKEN BioResource Research Centre, Cell No. RCB0535) were cultured in Dulbecco’s modified Eagle’s medium (DMEM) supplemented with 10% fetal bovine serum (FBS), penicillin (100 IU/ml) and streptomycin (100 IU/ml) at 37 °C in a humidified atmosphere with 5% CO_2_. HEK293T cells (Japanese Collection of Research Bioresources Cell Bank, Cell No. JCRB9068) were cultured in DMEM supplemented with 10% FBS, penicillin (100 IU/ml) and streptomycin (100 IU/ml) at 37 °C in 5% CO_2_. Authenticity of the cell lines was validated by short tandem repeat polymerase chain reaction. The absence of *mycoplasma* contamination was confirmed by nucleic acid staining.

### Vector constructs

iNOS complementary deoxyribonucleic acid was subcloned into the pCI vector. GFP-Rab5 in pcDNA3 and GST-Rab5Q79L, GST-Rab5 (wild) and GST-Rab5S34N pGEX-2T constructs were kindly provided by Dr Y. Yamamoto (Tokyo University of Agriculture). Rab5 was cloned into the pet30a vector to obtain His-Rab5. GST-Rab5C19A, GST-Rab5C63A, GST-Rab5C212A, GST-Rab5C213A, and GST-Rab5C212A/C213A in pGEX-2T were constructed by mutating GST-Rab5 (WT) in pGEX-2T using a Quik-Change Site-Directed Mutagenesis Kit (Agilent Technologies). The mutated Rab5 genes were then cloned into the pCMV-HA vector. The GST-R5BD vector was kindly provided by Dr G. Li (University of Oklahoma Health Science Center, Oklahoma City) (74). The pEGFP-C1-human RUTBC3 (also known as RabGAP-5) vector was purchased from RIKEN BRC (132). Hs.HRAS wild and Hs.HRAS C186S in pDonor-255 vectors were purchased from Addgene. To construct HA-Ras wild and HA-Ras C186S vectors, HRAS wild, or HRAS C186S was amplified using specific primers for HRAS and then inserted into the pCMV-HA vector.

### Antibodies

Antibodies were obtained from the following sources: anti-mouse HA and anti-rabbit HA (Sigma-Aldrich); anti-rabbit IgG-Alexa 555 and anti-rabbit IgG-Alexa 633 (Life Technologies); anti-mouse Rab5, anti-rabbit Rab5, anti-mouse GFP (Roche), and anti-rabbit GFP (Novus); anti-mouse IgG-horseradish peroxidase (HRP) and anti-rabbit IgG-HRP (IBL); anti-GST HRP conjugate (GE Healthcare); anti-mouse GAPDH (MBL); anti-mouse His (Sino Biological; and anti-rabbit iNOS (NOS2) (Santa Cruz Biotechnology). Antibody specificity was confirmed through experiments using overexpression systems, knockdown systems, or purified proteins.

### Transfection

RAW264 cells were transfected with plasmids using Lipofectamine 3000 (Life Technologies) and incubated for 3 h. The medium was then aspirated and replaced with fresh medium. The cells were once again transfected with plasmids using Lipofectamine 3000. HEK293 cells were transfected once using Lipofectamine.

### Phagocytosis assay

Transfected cells or cells treated with GSNO or L-NAME hydrochloride (hereafter abbreviated as L-NAME) in 96-well plates were preincubated with serum-free DMEM without phenol red for 1 h at 37 °C. The medium was then aspirated and 1 mg/ml pHrodo red-labeled *S. aureus* BioParticles (Life Technologies), a phagocytosis marker that was diluted with serum-free DMEM without phenol red, was added to the cells. The cells were incubated for the indicated times at 37 °C, and phagocytic uptake was analyzed by measuring fluorescence signal intensity with a SpectraMax m3 multimode microplate reader (Molecular Devices Japan) (excitation wavelength: 560 nm/fluorescence wavelength: 585 nm). Fluorescence microscopy observations were made under the same conditions.

### Western blotting

SDS-PAGE and Western blotting were performed using experimental conditions reported by Hagiwara (133, 134), except that a E-T520L e-PAGEL (5–20% gradient SDS-polyacrylamide gel, catalog number 2331830, ATTO Corporation) was used. The specific bands were quantified using ImageJ Fiji (https://imagej.net/software/fiji/downloads).

### Bacterial protein expression and purification

Bacterial expression and purification of GST-R5BD was performed as previously described (12, 15, 30). Purified GST-R5BD was stored at −80 °C until use.

Purified His-Rab5 was prepared using a standard procedure. In brief, the Rab5 in the pet30a vector was transformed into *E. coli* BL21-CodonPlus (DE3) RIL competent cells (Agilent Technologies) and protein expression was induced with 1 mM IPTG for 3 h at 37 °C. His-Rab5 was purified from bacterial cell extracts by passage over a His Trap HP column (GE Healthcare) and elution with imidazole according to the manufacturer's instructions. Desalting and buffer exchange into PBS was conducted with an Amicon Ultra device (Merck). Purified His-Rab5 was stored at −80 °C until use.

### Immunoprecipitation

Cells were lysed on ice in buffer (10 mM Tris, pH 7.6, 150 mM NaCl, 5 mM MgCl_2_, 1% Triton X-100) containing protease inhibitors and clarified by centrifugation. Cell lysates were incubated with an antibody against the indicated target protein with rotation for 1 h at 4 °C. Protein A-agarose beads were then added to the cell lysates and the mixtures were incubated for an additional 1 h at 4 °C. After washing, the immunoprecipitated proteins were dissolved in SDS-sample buffer and analyzed by Western blotting.

### Immunostaining

After washing with PBS, cells were fixed in 4% paraformaldehyde for 10 min, permeabilized with 0.1% Triton X-100 for 10 min, and blocked with blocking reagent N101 (NOF corporation) for 1 h at room temperature. The cells were then incubated with primary antibodies against the indicated target proteins, followed by incubation with the respective fluorescent secondary antibodies. After extensive washing, cells were mounted on coverslips using Shandon Immu-Mount (Thermo Fisher Scientific-JP). Cells were observed with confocal fluorescence microscopy TCS SP8 (Leica Microsystems), and images were analyzed using ImageJ Fiji.

### Detection of S-nitrosylation (Western blotting *via* biotin-switch assay)

A biotin-switch assay was carried out using an S-nitrosylated Protein Detection Kit (Cayman) according to the manufacturer’s instructions. Biotinylated proteins were precipitated with streptavidin agarose beads and Western blotting was performed to detect S-nitrosylated proteins using a specific primary antibody and an HRP-conjugated secondary antibody. ImmunoStar LD or ImmunoStarR Zeta (FUJIFILM Wako Pure Chemical Corp; catalog number 290-69904 and 295-72404, respectively) was used as the detection reagent, and proteins were detected with a chemiluminescence device. The specific bands were quantified using ImageJ Fiji.

### Detection of S-nitrosylation (fluorescence microscopy *via* biotin-switch assay)

A biotin-switch assay was carried out using an S-Nitrosylated Protein Detection Kit (Cayman) according to the manufacturer’s instructions. S-Nitrosylated proteins were visualized using fluorescein-conjugated avidin, and HA-Rab5 was detected using a specific-HA antibody (primary antibody) and a cy3-conjugated secondary antibody. After extensive washing, cells were mounted on coverslips using Shandon Immu-Mount (Thermo Fisher Scientific-JP). Cells were observed with confocal fluorescence microscopy TCS SP8 (Leica Microsystems), and images were analyzed using ImageJ Fiji.

### GST-R5BD pull-down assay

A GST-R5BD pull-down assay was performed as previously described (12, 15, 30). Briefly, glutathione Sepharose beads were coated with 30 μg of GST-R5BD. The beads were then incubated with lysates of freshly transfected HEK293T cells or purified His-Rab5 for 30 min at 4 °C. The beads were washed and subjected to Western blotting.

### GDP release assay and GTP-binding assay

A GDP release assay using mant-GDP and a GTP-binding assay using mant-GTP were performed as previously reported (89, 90, 135). Fluorescence measurements were made using a SpectraMax m3 multimode microplate reader (excitation wavelength: 355 nm/fluorescence wavelength: 448 nm).

### Subcellular fractionation

Cells expressing each of the HA-Rab5 mutants were homogenized in Hepes buffer (20 mM Hepes (pH 7.4), 100 mM NaCl, 5 mM MgCl_2_) containing protease inhibitors. The homogenates were centrifuged at 800*g* for 10 min at 4 °C to remove cell debris. The supernatant was further centrifuged at 200,000*g* for 30 min, yielding supernatant (S) and pellet (P) fractions. P was subsequently resuspended in Hepes buffer. S and P fractions were analyzed by Western blotting.

### Visualization of S-nitrosylated proteins

A biotin-switch assay was performed using an S-Nitrosylated Protein Detection Kit (Cayman) according to the manufacturer’s instructions. Cells were observed with confocal fluorescence microscopy, and images were analyzed using ImageJ Fiji (https://imagej.net/software/fiji/downloads).

### Observation of phagosome formation and phagocytosis assay using latex beads

RAW264 cells were incubated with 100 ng/ml LPS at 37 °C for 24 h, after which fluorescent latex beads were added and the cells were further incubated at 37 °C for 2 h (The ratio of cells to latex beads is 1:100). The cells were then immunostained as described above and observed using confocal fluorescence microscopy. For the phagocytosis assay using latex beads, RAW264 cells cultured in 6-well plates were incubated with 100 ng/ml LPS at 37 °C for 24 h. Fluorescent latex beads were then added, and the cells were further incubated at 37 °C for 2 h. The cells were collected and lysed on ice using PBS containing NP-40 (final concentration 0.3%). The lysates were transferred to a microplate. Fluorescence was measured using a microplate reader.

### *In vivo* Rab5 activity measurement and Rab5 S-nitrosylation analysis

Female mice (C57BL/6J JmsSlc) were purchased from Japan SLC, Inc. To detect Rab4 S-nitrosylation, mice were injected intravenously with LPS or LPS and L-NAME, and sacrificed 24 h later. The spleens were immediately collected and homogenized for analysis of Rab5 S-nitrosylation using an S-Nitrosylated Protein Detection Kit (Cayman Chemical) according to the manufacturer’s instructions and for measurement of Rab5 activity using a GST-R5BD pul- down assay as described above. These experiments were approved by the Animal Experiment Committee of the National Center for Geriatrics and Gerontology (Approval number 26-21-R1).

### *S. aureus* culture conditions

*S. aureus* ATCC 35896 was cultured aerobically in brain heart infusion (BHI) broth at 37 °C for 12 h. Bacterial cultures in the log phase of growth were then centrifuged for 15 min at 8500*g*, washed three times with PBS, and cultured aerobically on BHI agar plates at 37 °C for 12 h. The number of CFUs/ml was then determined.

### Clearance of *S. aureus* by intraperitoneal macrophage transfer

Donor female C57BL/6 mice were intraperitoneally injected with 4% thioglycolate (2 ml/mouse). Peritoneal macrophages collected 4 days later were cultured in 6-well plates with DMEM/F-12 containing 10% FBS with 4 × 10^6^ cells/well. Macrophages were treated with 1 mM L-NAME for 24 h and washed three times with PBS. Adherent cells were collected using Accutase (Nacalai Tesque). Host female C57BL/6 mice were intraperitoneally injected with *S. aureus* (3 × 10^9^ CFU/mice) together with the macrophages (4 × 10^6^ CFU/mice). After 6 h, *S. aureus* in the peritoneal lavage fluid was cultured on BHI agar and the number of CFU was determined. These experiments were approved by the Animal Experiment Committee of Tohoku University (Approval number 2014-037).

### Statistical analysis

Statistical analysis of results from experiments having two samples was conducted using an F-test followed by a *t* test. Statistical analysis of results from experiments having more than three samples was conducted using the Holm–Bonferroni method. *p* values ≤0.05 were considered significant.

## Data availability

All data are contained within the article. Further detailed information is available upon request from hagimako@unii.ac.jp.

## Conflict of interest

The authors declare that they have no conflicts of interest with the contents of this article.
